# Molecular and Metabolic Markers of Fructose Induced Hepatic Insulin Resistance in Developing and Adult Rats are Distinct and *Aegle marmelos* is an Effective Modulator

**DOI:** 10.1038/s41598-018-33503-x

**Published:** 2018-10-29

**Authors:** Jayachandran Nair, Thirumurthy Velpandian, Ujjalkumar Subhash Das, Prateek Sharma, Tapas Nag, Sandeep R. Mathur, Rajani Mathur

**Affiliations:** 1 Department of Pharmacology, Delhi Institute of Pharmaceutical Sciences and Research, Pushp Vihar, Sec III, MB Road, New Delhi, 110017 India; 2 Department of Ocular Pharmacology, Dr. R.P. Centre for Ophthalmic Sciences, All India Institute of Medical Sciences, Ansari Nagar East, Aurobindo Marg, New Delhi, 110029 India; 3 Department of Anatomy, All India Institute of Medical Sciences, Ansari Nagar East, Aurobindo Marg, New Delhi, 110029 India; 4 Department of Pathology, All India Institute of Medical Sciences, Ansari Nagar East, Aurobindo Marg, New Delhi, 110029 India

## Abstract

The time course of pathogenesis of fructose mediated hepatic insulin resistance (HepIR) is not well-delineated and we chronicle it here from post-weaning to adulthood stages. Weaned rats were provided for either 4 or 8 weeks, i.e., upto adolescence or adulthood, chow + drinking water, chow + fructose, 15% or chow + fructose, 15% + hydroalcoholic extract of leaves of *Aegle marmelos* (AM-HM, 500 mg/kg/d, po) and assessed for feed intake, fructose intake, body weight, fasting blood sugar, oral glucose tolerance test, HOMA-IR, insulin tolerance test and lipid profile. Activities of enzymes (glucose-6-phosphatase, hexokinase, phosphofructokinase, aldehyde dehydrogenase), hormones (leptin, ghrelin, insulin), insulin signaling molecules (Akt-PI3k, AMPK, JNK) hallmarks of inflammation (TNF-α), angiogenesis (VEGF), hypoxia (HIF-1), lipogenesis (mTOR) and regulatory nuclear transcription factors of *de novo* lipogenesis and hepatic insulin resistance gene (SREBP-1, FoxO1) that together govern the hepatic fructose metabolism, were also studied. The effect of fructose-rich environment on metabolic milieu of hepatocytes was confirmed using (human hepatocellular carcinoma) HepG2 cells. Using *in vitro* model, fructose uptake and glucose output from isolated murine hepatocytes were measured to establish the HepIR under fructose environment and delineate the effect of AM-HM. The leaves from the plant *Aegle marmelos* (L) Correa were extracted, fractionated and validated for rutin content using LC-MS/MS. The rutin content of extract was quantified and correlated with oral pharmacokinetic parameters in rat. The outcomes of the study suggest that the molecular and metabolic markers of fructose induced HepIR in developing and adult rats are distinct. Further, AM-HM exerts a multi-pronged attack by raising insulin secretion, augmenting insulin action, improving downstream signaling of insulin, reducing overall requirement of insulin and modulating hepatic expression of glucose transporter (Glut2). The butanol fraction of AM-HM holds promise for future development.

## Introduction

Metabolic syndrome and its prodrome hepatic insulin resistance (HepIR), are now increasingly evident in children and adolescents^1,2^. The scrooge behind onset of HepIR is now categorically linked to the rise in intake of fructose-rich diet such as soda, beverages, cakes, pastries, breakfast cereal^3^. Despite the tsunami of fructose induced HepIR in developing age-groups, the condition is not yet identified as a distinct pathological state but mere extension of the condition seen at adulthood. Consequently, the question that arises is that do the diagnostic molecular markers and management strategies that have been formulated to cater to adult subjects, hold true for developing age-groups, as well?

Thus, to address the query, it becomes imperative to (a) map the pathogenesis of the disease progression in developing age-groups and identify key molecular and metabolic markers; (b) develop management strategies that affect the identified markers to mitigate the disease. The answers to these queries are important as they can help to effectively diagnose, identify, and treat fructose induced HepIR in developing age-groups.

In order to determine the time course of metabolic changes in developing age-groups due to consumption of fructose-laden beverages and compare them against those manifested at adulthood, the present study was designed to include fructose as drinking solution (15%) in diet of weaned pups for either 4 or 8 weeks study duration, i.e, till they attained either adolescence (56 days old) or adulthood (84 days old). The effects have been confirmed using *in vitro* study where human hepatic cell carcinoma cells (HepG2) were grown in fructose-rich culture media.

Humans have long-standing knowledge and faith on the benefits of the medicinal plants as both food and medicine. *Aegle marmelos* (L) Correa (Family: Rutaceae), commonly known as Bael, is well documented in ancient literature as a natural remedy for many illnesses^4^. Rutin (Ru), a glycoside-flavonoid, has been identified as the main constituent of *A*. *marmelos* and attributed to many biological effects^5^. In the present study, the hydroalcoholic extract of leaves of *A*. *marmelos* (AM-HM) and its fractions have been prepared, standardized by LC-MS/MS in terms of their Ru content and investigated for pharmacodynamic effects in models of fructose induced HepIR. Further, for the first time the oral pharmacokinetics of *A*. *marmelos* is elucidated.

Thus, we report here results from an exhaustive investigation on the pharmacodynamic effects of *A*.*marmelos* along with their pharmacokinetic correlation, on molecular and metabolic markers of fructose induced HepIR in different age groups.

## Results

### LC-MS/MS method validation and standardization of AM-HM and its fractions

**Figure 1:**
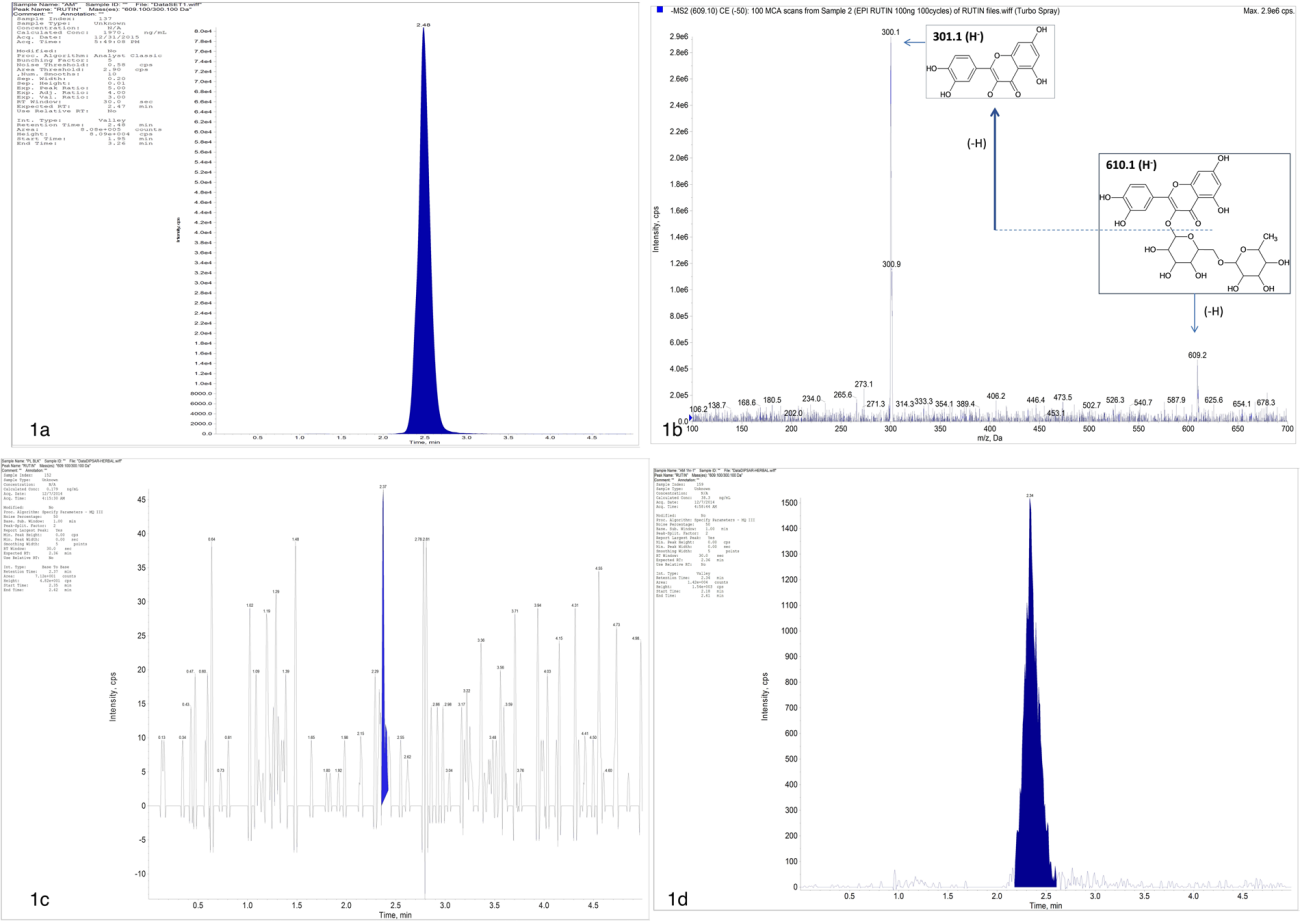
Schematic fragment ion intensity chromatogram of Rutin (Ru), its quantification in AM-HM and plasma using LC-MS/MS. Chromatogram of Ru present in hydro-alcoholic extract of leaves of *Aegle marmelos* (AM-HM,1 mg/ml) and standard Ru used for MS2 fragmentation (**a**,**b**). Representative chromatogram of blank human plasma wherein no peak of Ru could be detected (**c**). Well separated, finely resolved, sharp peak of Ru could be detected at 1 h time point (**d**) in plasma following administration of AM-HM (500 mg/kg, po) to normal rat.

The developed method for estimation of Ru was found robust and linear in the range of 3.9–500 ngmL^−1^ with regression coefficient of 0.998 (Fig. 1a). The fragmentation pattern for Ru using (-ve) ESI- MS2 product ion mode is represented (Fig. 1b). The slope of regression line was 47.662 with y-intercept at 621.43 (y = 47.662x + 621.43).

The LOD for the method was 0.7911 ngmL^−1^ while the LOQ was found to be 2.397 ngmL^−1^. The method was found accurate with good recovery and no matrix effect for Ru at 7.8, 15.6 and 500 ngmL^−1^. The average recovery (%) and matrix effect (%) were 91.863 ± 15.923, 100.234 ± 17.272, 109.483 ± 3.557 and 102.117 ± 12.852, 106.754 ± 26.272, 95.343 ± 1.750, respectively. For interday and intraday studies, the CV (%) of the method was 6.33, 4.38, 2.71 and 27.22, 11.34, 1.128, respectively. For interday and intraday studies, the accuracy (%) of the method was 86.866, 91.944, 96.644 and 83.418, 91.5242, 104.088, respectively. The above values were within the acceptable range and demonstrated repeatability.

The Ru content in AM-HM, AM-C, AM-H, AM-EA, AM-B and AM-A was 1.97, 0.202, 0.123, 0.684, 7.210, 0.909 µg/mg, respectively.

### *In vivo* oral pharmacokinetic studies of AM-HM

The study was executed to quantify Ru in the blood and plot its pharmacokinetic profile after oral administration of AM-HM (Fig. 1c). The Ru concentration at 0.08 min, 0.5, 1, 2, 4, 24 h was 0.697 ± 0.370, 12.4 ± 1.310, 16.806 ± 8.616, 8.013 ± 0.518, 2.52 ± 0.103, 0.939 ± 0.048 ngmL^−1^, respectively (Fig. 1d). The pharmacokinetic parameters-Tmax, Cmax, t_1/2_, AUC and AUC_0-α_ were 1.5 ± 0.866 h, 15.3 ± 8.022 ngmL^−1^, 0.077 ± 0.019 h, 60.612 ± 16.558 hng^−1^and 74.268 ± 18.502 hng^−1^, respectively.

### Effect of AM-HM on feed and fructose/water intake, body weight and caloric intake

#### Study I

**Figure 2:**
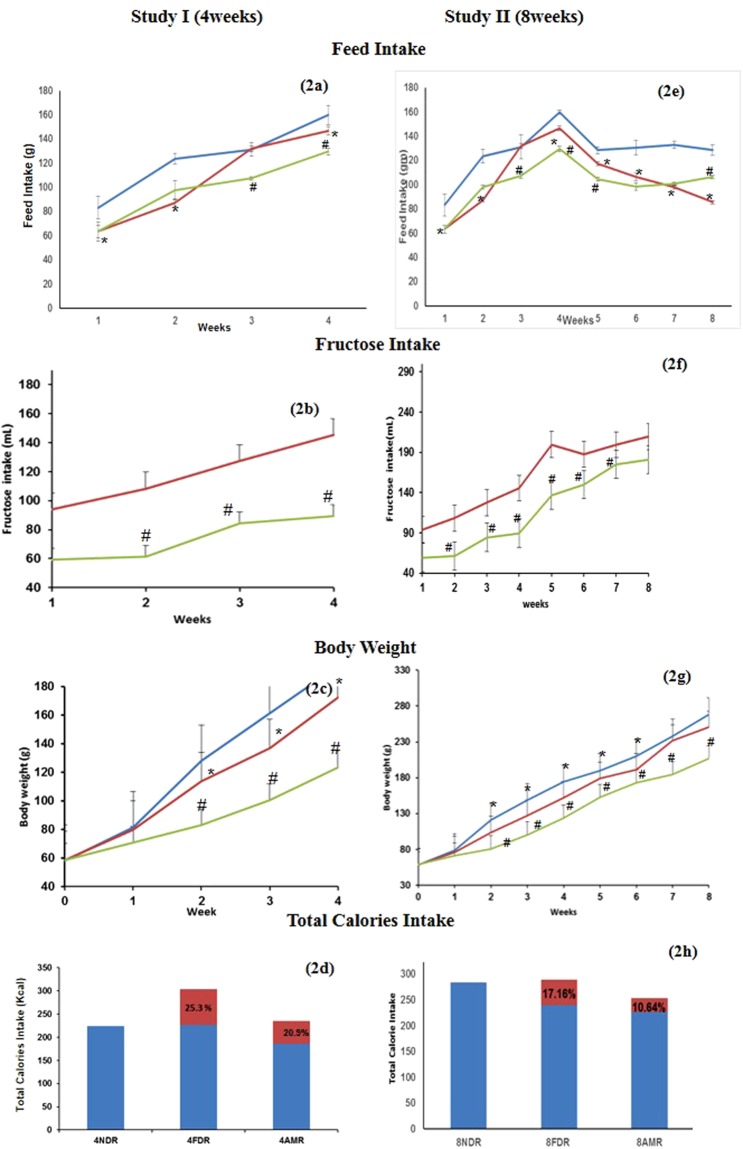
Comparison of weekly feed intake, fructose intake, body weight and total calorie intake, after 4 weeks(study I) and 8 weeks (study II) of fructose ingestion. The feed intake, fructose intake, body weight and total calorie intake at developing (**a–d**) and adulthood (**e–h**) stages in normal (blue line), fructose-control (red line) and AM-HM (green line) treated groups are represented. The feed and fructose intake were highest in NDR and lowest in AMR in studies I and II. The weight gain pattern was parallel in all the three groups with lowest gain in AMR and highest in NDR in studies I and II. The total caloric intake, in study I, was highest in FDR with about 25% sourced from fructose. In study II, the total caloric intake, was equal in NDR and FDR with about 17% sourced from fructose in latter. All values are mean ± SEM; (n = 6), *p < 0.05 *vs* NDR, ^#^p < 0.001 *vs* FDR

The feed intake of 4NDR was significantly higher (p < 0.05) than 4FDR and 4AMR over the entire study duration (Fig. 2a). At the 3^rd^ and 4^th^ week of the study, the feed intake of 4FDR was significantly greater (p < 0.05) than 4AMR. The fructose intake was significantly reduced (p < 0.05) in 4AMR as compared to 4FDR (Fig. 2b). The body weight gain pattern of 4FDR was parallel and rightward of 4NDR and significantly lower (p < 0.05) at 2^nd^, 3^rd^ and 4^th^ week of the study (Fig. 2c). The body weight of 4AMR was significantly lower (p < 0.05) than 4FDR at 2^nd^, 3^rd^ and 4^th^ week of the study (Fig. 2c). The fraction of the total calories derived from fructose in 4FDR and 4AMR was 25.34 and 20.9%, respectively (Fig. 2d).

#### Study II

At all study time points, the feed intake of 8FDR was recorded significantly lower (p < 0.05) than 8NDR (Fig. 2e). The fructose intake was significantly high (p < 0.05) in 8FDR as compared to 8AMR (Fig. 2f). The body weight gain of 8FDR was significantly lower (p < 0.05) than 8NDR (Fig. 2g). The weight gain of 8AMR was significantly lower than 8FDR. The fraction of the total calories derived from fructose over 8-week study period was about 17.16 and 10.64% in 8FDR and 8AMR, respectively (Fig. 2h).

### Effect of AM-HM on fasting blood glucose, OGTT, HOMA-IR and ITT

#### Study I

**Figure 3:**
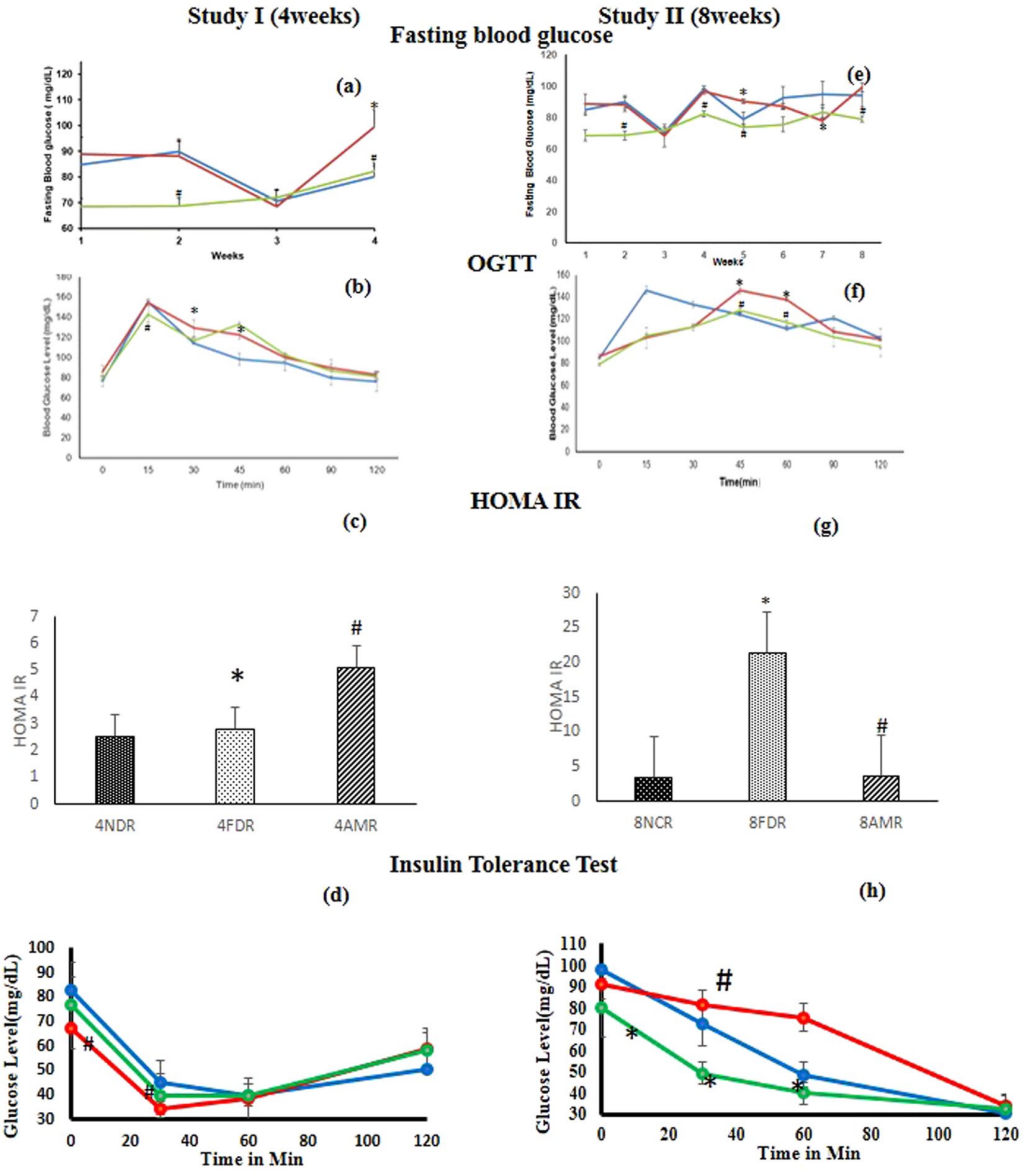
Comparison of weekly fasting blood glucose levels (FBG), presentation of oral glucose tolerance test (OGTT), homeostatic model assessment-insulin resistant (HOMA-IR) and insulin tolerance test (ITT) after 4 weeks(study I) and 8 weeks (study II) of fructose ingestion. The FBG, OGTT, HOMA-IR, ITT at developing (**a–d**) and adulthood (**e–h**) stages in normal (blue line), fructose-control (red line) and AM-HM (green line) treated groups are represented. The FBG was lowest in 4AMR and 8AMR. In studies I and II, the AUC-OGTT was highest in FDR as compared to NDR. HOMA-IR, an indicator of feedback loop between the liver and the β-cell, was significantly elevated in 4FDR and 4AMR, as compared to 4NDR and 4FDR, respectively. In study II, the significantly raised HOMA-IR value in 8FDR as compared to 8NDR was reverted in 8AMR. In ITT, the simulated hypoglycemic response in 4NDR, 4AMR was parallel. The hypoglycemia nadir in 4FDR was at the same time point as 4NDR, albeit significantly steeper, implying impairment of HPA axis. In study II, at adulthood, the animals in 8FDR recorded a delayed and attenuated response to insulin in terms of lowering blood glucose levels, evidencing an IR state, which was overcome in 8AMR. All values are mean ± SEM; (n = 6), *p < 0.05 *vs* NDR, ^#^p < 0.001 *vs* FDR.

At the end of 4^th^ week of study, the FBG of 4FDR was significantly higher (p < 0.05) than 4NDR (Fig. 3a). The 4AMR recorded significant fall (p < 0.05) in FBG at the 4^th^ week of the study as compared to 4 FDR (Fig. 3a). The OGTT curve of 4FDR recorded a peak at 15 min time point and remained significantly elevated (p < 0.05) up to 45 min time point as compared to 4NDR (Fig. 3b). The rise in blood glucose level in 4AMR in response to oral glucose was lower than 4FDR (Fig. 3b). The HOMA-IR value of 4NDR, 4FDR and 4AMR was 2.502 ± 0.713, 2.808 ± 0.915 and 5.100 ± 0.703, respectively (Fig. 3c). In the ITT, the AUC of 4NDR, 4FDR and 4AMR was 2885 ± 58.81, 2021.25 ± 54.06 and 2379 ± 76.77, respectively (Fig. 3d).

#### Study II

At the 5^th^ week of the study, the FBG in 8FDR was significantly higher (p < 0.05) than 8NDR (Fig. 3e). The FBG in 8AMR was significantly lower than 8FDR at each time point of the study, except at the 7^th^ week (Fig. 3e). In the OGTT, the 8NDR recorded a sharp rise in blood glucose level at 15 min post glucose feeding (Fig. 3f). The 8FDR recorded a significant rise (p < 0.05) in blood glucose level between 45 and 60 min post glucose feeding as compared to 8NDR that was significantly attenuated (p < 0.05) in 8 AMR (Fig. 3f). The HOMA-IR value of 8NDR, 8FDR and 8AMR was 3.441 ± 1.719, 21.290 ± 2.053, 3.543 ± 1.071, respectively (Fig. 3g). In the ITT, the AUC of 8NDR, 8FDR and 8AMR was 4925 ± 62.52, 6175 ± 60.25 and 3500 ± 34.77, respectively (Fig. 3h). The AUC of 8FDR was significantly elevated, as compared to 8NDR but was reverted in 8AMR.

### Effect of AM-HM on visceral weight and biochemical estimations

#### Study I

At the end of the study, the weight of liver in 4FDR was about 55% greater than 4NDR (p < 0.05) (Table 1). The plasma leptin and ghrelin levels were 3 and 74 fold lower in 4FDR (p < 0.05) as compared to 4NDR but raised by a factor of 1.5 and 70 in 4AMR as compared to 4FDR, respectively (Table 1). The insulin level was elevated 2-fold in 4AMR as compared to 4NDR (Table 1). Following intake of fructose for 4 weeks, the TG and LDL values in 4FDR were raised by about 26 and 68%, respectively, in comparison to 4NDR. The TG and LDL levels were reduced in 4AMR by 30 and 68%, respectively (Table 1). Serum uric acid levels were 1.5 fold higher in 4FDR but reduced significantly (p < 0.05) in 4AMR (Table 1). The activity of AST and ALT was reduced in the 4FDR but raised in 4AMR (Table 1).

**Table 1:** Comparison of visceral weight, lipid profile, liver function test, activities of pro-inflammatory markers and enzymes of glycolysis and gluconeogenesis after 4 weeks(study I) and 8 weeks (study II) of fructose ingestion.

Parameters	4NDR	4FDR	4AMR	8NDR	8FDR	8AMR
Heart(g)	0.649 ± 0.032	0.752 ± 0.103	1.232 ± 0.123^#^	0.934 ± 0.133	0.998 ± 0.054	0.711 ± 0.046^#^
Kidney(g)	0.626 ± 0.098	0.759 ± 0.105	1.245 ± 0.146^#^	0.865 ± 0.060	0.888 ± 0.067	0.757 ± 0.090
Liver(g)	4.750 ± 0.543	7.367 ± 1.149*	6.467 ± 0.594^#^	6.776 ± 1.191	8.162 ± 0.534*	6.800 ± 0.083^#^
Plasma Leptin level(pg/ml)	1165.233 ± 455.652	466.76 ± 322.069	738.3 ± 183.785	266.266 ± 30.157	3413.933 ± 36.081*	3249.1 ± 47.113^#^
Plasma Gherlin (ng/mL)	455.071 ± 66.235	6.140 ± 0.381*	427.149 ± 14.386#	580.573 ± 1.923	746.260 ± 17.745*	529.323 ± 14.790^#^
**Insulin**						
(µIU/mL)	11.967 ± 3.597	13.278 ± 4.448	26.147 ± 0.189^#^	14.754 ± 7.195	102.250 ± 8.995*	18.196 ± 4.550^#^
Total cholesterol(mg/dl)	112.666 ± 17.351	99.666 ± 5.085	117.333 ± 12.209^#^	64.666 ± 2.886	65 ± 7	72.666 ± 13.051
TG(mg/dl)	79.666 ± 18.980	91.333 ± 8.066*	63.333 ± 24.695^#^	39.666 ± 9.712	188 ± 74*	59 ± 9.643^#^
HDL(mg/dl)	74.666 ± 3.141	69.333 ± 7.174*	78 ± 7.797^#^	45 ± 2	40.333 ± 3.214*	47.666 ± 8.504^#^
LDL(mg/dl)	22.066 ± 13.458	37.733 ± 1.342*	12.4 ± 1.350^#^	11.733 ± 2.995	13.2 ± 11.301	13.2 ± 4.703^#^
VLDL(mg/dl)	15.933 ± 3.796	18.266 ± 1.613	46.066 ± 4.939^#^	7.933 ± 1.942	37.6 ± 14.8*	11.8 ± 1.928^#^
Serum Uric acid (n mol/mL)	1346.875 ± 70.374	2100 ± 59.844*	1643.75 ± 40.799^#^	1162.5 ± 88.388	2381.25 ± 35.355*	1834.375 ± 61.871^#^
Liver Aspartate Transaminase (IU/L)	321.950 ± 132.944	189.218 ± 0.719*	158.677 ± 219.528^#^	124.097 ± 14.599	255.755 ± 52.986*	98.506 ± 44.211^#^
Liver Alanine Transaminase (IU/L)	491.975 ± 28.170	398.124 ± 26.878*	494.058 ± 83.987^#^	166.899 ± 60.341	396.827 ± 17.419*	151.93 ± 62.924^#^
Protein Level (μg/ml)	567.7 ± 282.962	606.033 ± 98.398*	464.700 ± 254.242^#^	1401.367 ± 24.041	1226.367 ± 18.384*	1357.033 ± 24.006^#^
HIF 1α (ng/ml)	0.334 ± 0.034	0.377 ± 0.025*	0.129 ± 0.144^#^	0.254 ± 0.032	2.312 ± 1.766*	0.349 ± 0.029^#^
VEGF (pg/ml)	2387 ± 94.280	822 ± 10.066*	437 ± 89.566^#^	500.333 ± 4.714	1068.667 ± 21.213*	515.333 ± 16.499^#^
TNF α (pg/ml)	14823 ± 38.890	17570.5 ± 49.497*	14948 ± 215.667^#^	14700.5 ± 247.487	15658 ± 24.748*	15155.5 ± 63.639
Glycogen (mg of glycogen/100 g tissue)	23.560 ± 10.368	84.261 ± 78.347*	36.742 ± 19.761^#^	57.5 ± 14.840	174.488 ± 15.918*	53.693 ± 7.645^#^
G6Pase (ng/10 mg)	47.095 ± 14.113	54.324 ± 23.869*	144.655 ± 76.867^#^	153.507 ± 80.693	161.947 ± 95.923*	129.494 ± 6.628^#^
FBPase (ng/10 mg)	29.897 ± 17.189	3.061 ± 16.182*	23.609 ± 22.775^#^	42.015 ± 16.006	49.808 ± 33.342*	56.492 ± 3.499 ^#^
HK (IU/mL)	13.333 ± 0.648	17.729 ± 21.419*	21.458 ± 22.214^#^	20 ± 7.542	4.229 ± 1.207*	24.479 ± 3.388^#^
LDH (μMol)	0.531 ± 0.180	3.406 ± 0.142*	1.289 ± 0.205^#^	1.345 ± 0.159	3.401 ± 0.277	0.423 ± 0.795
ALDH (IU/mL)	99.516 ± 46.112	112.086 ± 104.722*	162.497 ± 94.107^#^	81.636 ± 21.590	127.686 ± 57.405*	65.633 ± 20.329^#^
ALK(IU/mL)	10.566 ± 7.841	50.215 ± 4.875*	21.550 ± 13.469^#^	22.387 ± 8.945	33.616 ± 11.442*	29.571 ± 7.371^#^
PFK (ng/ml)	12.761 ± 2.104	18.214 ± 3.552*	10.261 ± 2.205^#^	19.357 ± 4.865	12.809 ± 0.353*	12 ± 0.353

The rise in weight of liver, insulin concentration, triglyceride level, glycogen content, pro-inflammatory markers after fructose ingestion, is phenomenal at developing than at adulthood stage. All values are mean ± SEM; (n = 6), *p < 0.05 *vs* NDR, #p < 0.001 *vs* FDR.

#### Study II

After eight weeks of fructose intake, the weight of liver in 8FDR was about 20% greater than 8NDR (Table 1). The plasma leptin and ghrelin levels were raised by about 7 and 1.5 fold in 8FDR but decreased significantly (p < 0.05) in 8AMR (Table 1). The insulin level was elevated 8-folds in 8FDR as compared to 8NDR and was reverted in 8AMR (Table 1). The TG level was elevated by 4 times in 8FDR as compared to 8NDR but significantly lowered (p < 0.05) in 8AMR (Table 1). The serum uric acid level was raised by 2-fold in the 8FDR as compared to 8NDR that was significantly reduced in the 8AMR (Table 1). The levels of liver aspartate transaminase and alanine transaminase were significantly increased (p < 0.05) in 8FDR as compared to 8NDR and the trend was significantly (p < 0.05) reversed in 8AMR (Table 1). The HIF-1α level was raised by 10-fold in 8FDR as compared to 8NDR but reverted in 8AMR (Table 1). The serum level of VEGF that was raised by over 2-fold in 8FDR as compared to 8NDR was reversed in 8 AMR (Table 1). The level of TNFα was raised significantly (p < 0.05) in 8FDR as compared to 8NDR (Table 1).

### Effect of AM-HM on glycogen and hepatic enzymes

#### Study I

A significant four-fold increase in glycogen level in liver tissue was recorded in 4 FDR as compared to 4 NDR but was halved in 4AMR as compared to 4FDR (Table 1). The activity of G6Pase was significantly (p < 0.05) raised in 4FDR as compared to 4NDR and further raised by 3 fold in 4 AMR as compared to 4FDR (Table 1). The FBPase activity was significantly reduced by 10-fold in 4FDR as compared to 4NDR that was reverted in 4AMR (Table 1). The activity of HK was significantly (p < 0.05) raised in 4FDR as compared to 4NDR (Table 1). The 4AMR recorded elevated activity of HK as compared to 4FDR (Table 1). The activity of LDH, ALDH, ALK and PFK was raised by 7, 1.1, 5 and 1.5-folds, respectively, in 4FDR as compared to 4NDR (Table 1). The level of ALK was halved in 4AMR as compared to 4FDR (Table 1).

#### Study II

A significant three-fold increase in glycogen level in liver tissue was recorded in 8FDR as compared to 8NDR but was significantly reduced (p < 0.05) to normal in 8AMR (Table 1). The activity of G6Pase was raised in 8FDR as compared to 8NDR and reduced in 8AMR (Table 1). The FBPase activity was raised in 8FDR as compared to 8NDR that was further elevated significantly in 8AMR (Table 1). The HK activity was significantly (p < 0.05) reduced by five-fold in 8FDR as compared to 8NDR and elevated by 6-fold in 8AMR as compared to 8FDR (Table 1). The activity of LDH, ALDH, and ALK in 8FDR was higher by 3, 1.5 and 1.5 folds, respectively, as compared to 8NDR but reverted in 8AMR (Table 1). The PFK activity was significantly (p < 0.05) reduced in 8FDR as compared to 8NDR (Table 1). The levels of ALK, were increased in the 8FDR but reduced by the 8AMR (Table 1).

### Effect of AM-HM on insulin signaling cascade-Akt, p-try-STAT-3, JNK1/2, AMPK 1 α, FoxO1, SREBP1-c

**Figure 4:**
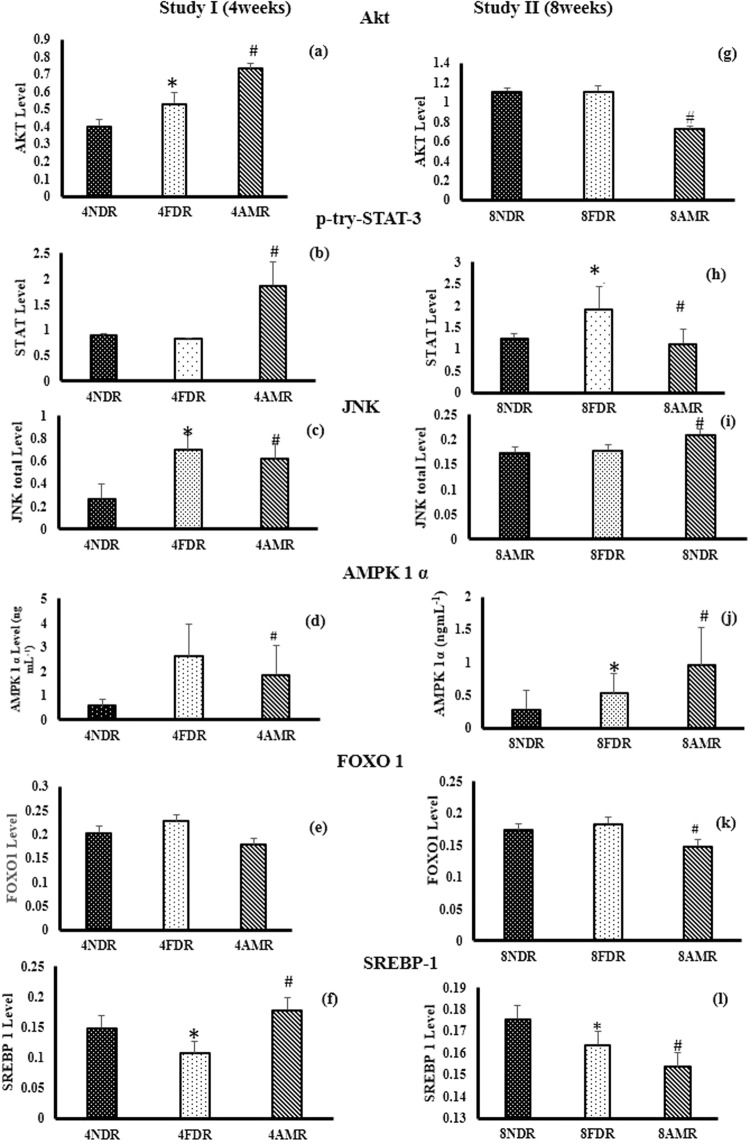
Effect of AM-HM on messengers of hepatic insulin signaling cascade-Akt, signal transducer and activator of transcription 3 (p-tyr-STAT-3), c-Jun N-terminal kinase (JNK1/2), 5′ AMP-activated protein kinase (AMPK-1α), Forkhead box protein O1 (FoxO1), sterol regulatory element-binding protein 1c (SREB1c). In study I, the Akt levels were significantly raised in 4FDR and 4AMR implying activation of insulin mediated glycolysis and glycogenesis pathways to siphon fructose. In study II, the unaltered levels of Akt in 8FDR, is suggestive of defect in insulin downstream signaling. In 8AMR, the downstream signaling sensitivity was significantly restored which coordinated with reduced insulin levels. The STAT-3 levels were significantly raised in 4AMR as compared to 4FDR. Chronic fructose ingestion significantly raised STAT-3 levels in 8 FDR as compared to 8NDR. At adulthood, 8AMR recorded significant reduction of STAT-3 as compared to 8FDR. The negative regulator of insulin signaling cascade, JNK, was significantly raised in 4FDR but reduced in 4AMR. At adulthood, the JNK level was not significantly different in 8FDR from 8NDR. The AMPK activity was significantly raised in 4AMR, that is indicative of fructose-induced ATP depleted state that was reversed in 4AMR but not in 8AMR. The 4AMR and 8AMR recorded significantly reduced FoxO1 levels as compared to 4FDR and 8FDR, respectively. The level of SREBP-1c were significantly reduced in 4FDR in consonance with reduced total cholesterol level. Chronic fructose ingestion, significantly reduced SREBP-1c levels in 8FDR as compared to 8NDR that was also reflected in total cholesterol levels. The 8AMR recorded significantly lower concentrations of SREBP-1c as compared to 8FDR.

The level of Akt protein was significantly elevated (p < 0.05) in 4FDR as compared to 4NDR that was further raised significantly (p < 0.05) in 4AMR (Fig. 4a). The level of p-try-STAT-3 protein was raised significantly (p < 0.05) in 4AMR as compared to 4FDR (Fig. 4b). The mean concentration of phosphorylated JNK1/2 in 4FDR was raised over three times (p < 0.05) as compared to 4NDR (Fig. 4c). The level of AMPK-1-α protein was significantly elevated (p < 0.05) in 4FDR as compared to 4NDR that was reduced in 4AMR (Fig. 4d). The transcription factor FoxO1 was marginally elevated in 4FDR, as compared to 4NDR and reduced in 4AMR (Fig. 4e). There was a significant reduction in the levels of the transcription factor SREBP-1c in 4FDR as compared to 4NDR, that was elevated by 4AMR (Fig. 4f).

#### Study II

The level of Akt protein was reduced by 40% in 8AMR as compared to 8FDR (Fig. 4g). The level of p-try-STAT-3 protein was 2-fold in 8FDR as compared to 8NDR that was significantly reduced (p < 0.05) in 8AMR (Fig. 4h). The mean concentration of phosphorylated JNK1/2 in 8AMR was significantly elevated as compared to 8NDR (Fig. 4i). The level of AMPK-1-α protein was significantly elevated (p < 0.05) in 8FDR as compared to 8NDR that was further significantly elevated (p < 0.05) in 8AMR (Fig. 4j). The transcription factor FoxO1 was significantly (p < 0.05) reduced in 8AMR as compared to 8AMR (Fig. 4k). There was a significant reduction (p < 0.05) in the levels of the transcription factor SREBP-1c in 8FDR as compared to 8NDR (Fig. 4l).

### Effect of AM-HM on hepatic GLUT 2 protein expression

#### Study I

**Figure 5:**
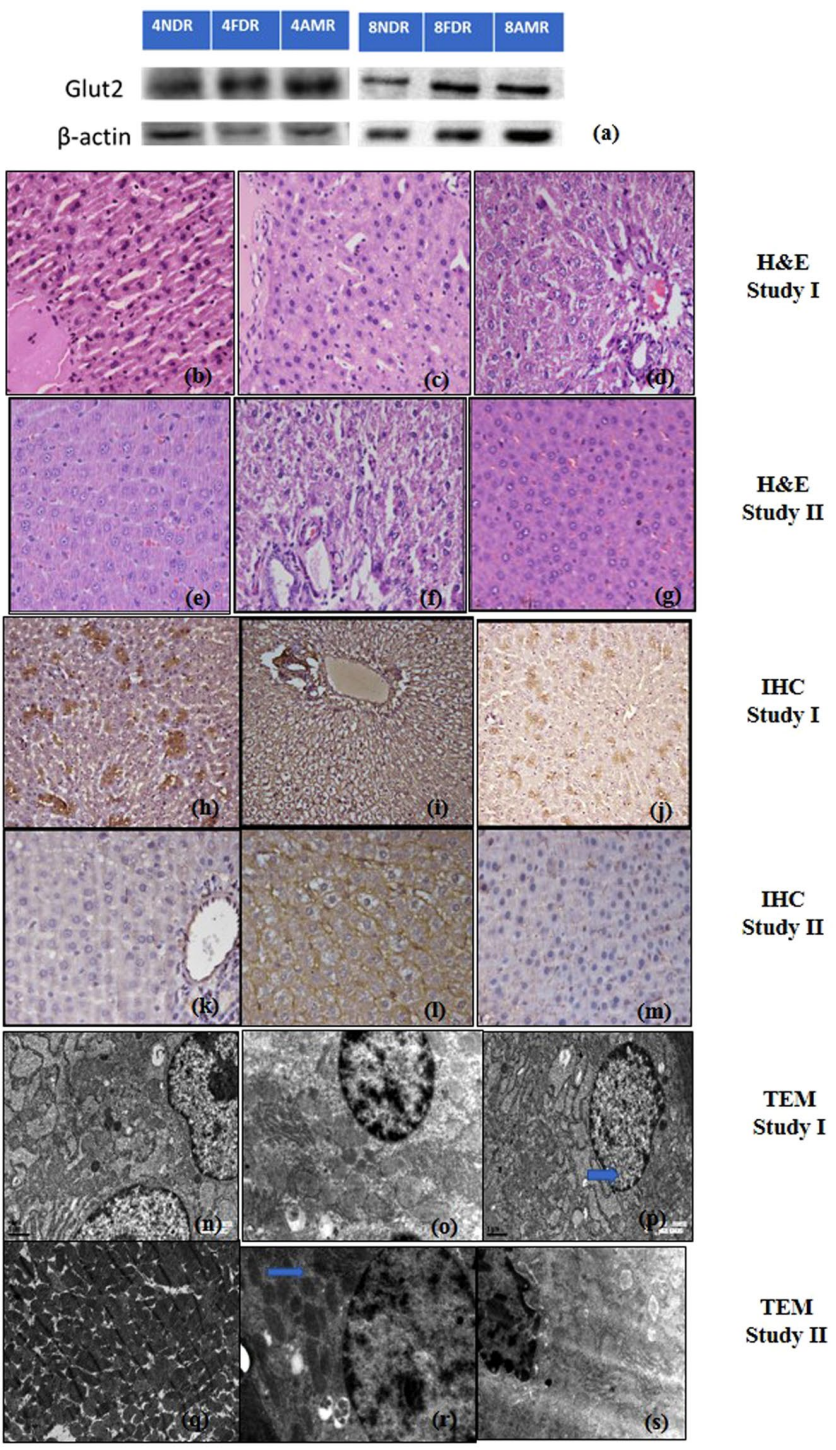
Comparative hepatic photomicrographs of western blot of glucose transporter (Glut2) protein, histopathology of parenchyma sections, immunohistochemistry of GLUT 2 protein and Transmission Electron Microscopy of hepatocytes, after 4 weeks(study I) and 8 weeks (study II) of fructose ingestion. The hepatic photomicrographs of western blot of glucose transporter (Glut2) protein, histopathology of parenchyma sections(H&EX200), immunohistochemistry of GLUT 2 protein and Transmission Electron Microscopy of hepatocytes in normal, fructose-control and AM-HM treated groups documents the histological, cellular and sub-cellular morphological changes brought about by fructose at developing (**a–d,h–j,n–p**) and adulthood (**a,e,f,g,k–m,q–s**) stages. The full-length blots/gels are presented in Supplementary Figures.

After four weeks of fructose (15%) intake, the mean densiometric value of the hepatic GLUT2 expression in 4NDR, 4FDR and 4AMR was 28173.58 ± 5698.674, 30672.90 ± 7623.908 and 24188.87 ± 5191.206, respectively (Fig. 5a).

#### Study II

The mean densiometric value of the hepatic GLUT2 expression in 8NDR, 8FDR, and 8AMR was 13587.41 ± 7627.366, 15071.87 ± 7495.332 and 19685.87 ± 8764.027, respectively (Fig. 5a).

### Effect of AM-HM on hepatic histology

#### Study I

The 4FDR recorded micro- and macrovesicular fatty changes of the hepatocytes but no apparent infiltration of markers for inflammatory cells, necrosis or fibrosis were observed. The 4AMR recorded protection against the fatty changes of hepatocytes observed in 4FDR. The histological architecture of liver section of 4AMR showed markedly reduced lobular pattern with mild degrees of fatty changes (Fig. 5b–d).

#### Study II

The 8FDR recorded fatty changes of the hepatocytes with infiltration of inflammatory cells. There was evidence of necrosis and fibrosis in 8FDR. The 8AMR recorded protection against the fatty changes of hepatocytes observed in 8FDR (Fig. 5e–g).

### Immunohistochemistry of hepatic GLUT2 protein

#### Study I

The Glut 2 protein was positive at the cell membranes of perivenular hepatocytes, in 4FDR hepatocytes but was reduced in 4AMR (Fig. 5h,i).

#### Study II

The Glut 2 protein was positive at the cell membranes of perivenular hepatocytes, in 8FDR hepatocytes but was decreased in 8AMR (Fig. 5k–m).

### Transmission electron microscopy (TEM) of liver

#### Study I

The TEM images of liver sections do not show major changes in the hepatic fragments. Cytosolic structure such as glycogen granules, were apparent in the 4FDR but not obvious in 4AMR (Fig. 5n–p).

#### Study II

The TEM images of liver sections from 8FDR, show densely localized vacuoles, and glycogen granules, that were sparingly present in the 8AMR (Fig. 5n–p).

### Effect of AM-HM on glycogen level in HepG2 cell line grown in fructose rich medium

**Figure 6:**
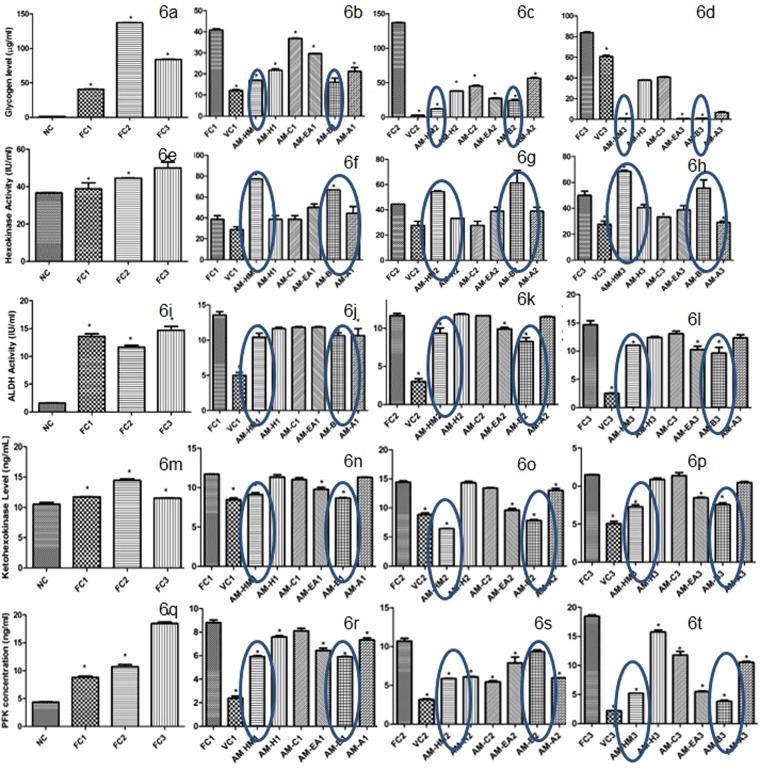
Effect of AM-HM on the milieu of sugar metabolizing enzymes of HepG2 when they are grown in fructose-rich media. Treatment of HepG2 cells grown in media either without glucose (NC) or with 0.55 mM fructose(FC1) or with 1 mM fructose (FC2) or with 1 mM fructose + 0.1 µM Insulin (FC3) with AM-HM (75 µg/ml), AM-H (75 µg/ml), AM-C (75 µg/ml), AM-EA (75 µg/ml), AM-B (75 µg/ml) or AM-A (75 µg/ml) for 48 hours affected the glycogen content (µg/ml), activities of glycolytic enzymes-hexokinase (IU/ml), aldehyde dehydrogenase(IU/ml), ketohexokinase (ng/ml) and irreversible regulator of glycolysis-phosphofructokinase (ng/ml). The AM-HM and AM-B reduced the glycogen level in FC1 & FC2 and the effect was augmented by insulin in FC3. The AM-HM and AM-B potentiated the action of HK in FC1 & FC2 and the effect was augmented by insulin in FC3. The AM-HM and AM-B reduced the activities of ALDH, KHK and PFK in FC1 & FC2 and the effect was augmented by insulin in FC3. Each value is the mean ± SD of n = 3. *p < 0.05 *vs* control using student-*t*-test.

The glycogen level rose significantly (p < 0.05) in FC1 and FC2 as compared to NC. The glycogen level in FC3 was significantly lower than FC2 (Fig. 6a). Significant reduction (p < 0.05) in glycogen level was recorded in AM-HM1, AM-H1, AMC1, AM-EA1, AM-B1 and AM-A1 as compared to FC1 (Fig. 6b). Similarly, AM-HM and its fractions significantly reduced (p < 0.05) glycogen level in FC2 and FC3 environment as compared to respective control (Fig. 6c–d).

### Effect of AM-HM on *HK* activity in HepG2 cell line grown in fructose rich medium

The hexokinase activity was significantly (p < 0.05) raised in FC1, FC2 and FC3 as compared to NC (Fig. 6e). The hexokinase activity was further raised significantly (p < 0.05) in AM-HM1 and AM-B1 (p < 0.05) as compared to FC1 (Fig. 6f). The hexokinase activity was raised significantly in AM-HM2 and AM-B2 (p < 0.05) as compared to FC2 (Fig. 6g). The hexokinase activity was further raised significantly in AM-HM3 and AM-B3 (p < 0.05) as compared to FC3 (Fig. 6h).

### Effect of AM-HM on ALDH activity in HepG2 cell line grown in fructose rich medium

The ALDH activity was increased significantly (p < 0.05) in FC1– FC3 groups as compared to NC (Fig. 6i). Significant reduction in ALDH (p < 0.05) by AM-HM1 and AM-B1 was recorded as compared to FC1 (Fig. 6j). Similarly, significant reduction (p < 0.05) in ALDH activity was recorded by AM-HM2 and AM-B2 as compared to FC2 and AM-HM3 and AM-B3 as compared to FC3 (Fig. 6k,l).

### Effect of AM-HM on KHK activity in HepG2 cell line grown in fructose rich medium

The activity of KHK was significantly elevated (p < 0.05) in FC1– FC3 groups as compared to NC (Fig. 6m). Significant reduction (p < 0.05) in KHK activity was recorded in AM-HM1, AM-EA1 and AMB1 as compared to FC1 (Fig. 6n). Similarly, significant reduction (p < 0.05) in KHK activity was recorded by AM-HM2, AM-EA2 and AM-B2 as compared to FC2 and AM-HM3, AM-EA3 and AM-B3 as compared to FC3 (Fig. 6o,p).

### Effect of AM-HM on PFK activity in HepG2 cell line grown in fructose rich medium

The PFK concentration was increased significantly (p < 0.05) in FC1–FC3 groups as compared to NC (Fig. 6q). Significant reduction (p < 0.05) in PFK activity was recorded in AM-HM1, AM-EA1, AM-B1 as compared to FC1 (Fig. 6r). Similarly, significant reduction (p < 0.05) in PFK activity was recorded in AM-HM2, AM-EA2 and AM-B2 as compared to FC2 and AM-HM3, AM-EA3 and AM-B3 as compared to FC3 (Fig. 6s,t).

### Effect of AM-HM on phosphatidylinositol-4,5-bisphosphate 3-kinase (PI3K) in HepG2 cell line grown in fructose rich medium

**Figure 7:**
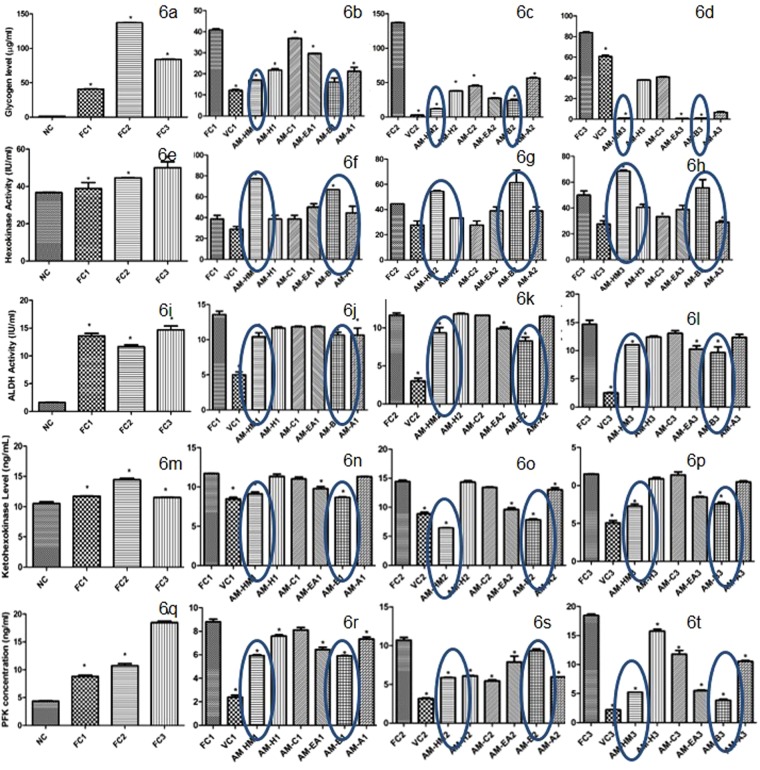
Effect of AM-HM on the molecular markers of IR in HepG2 when they are grown in fructose-rich media. Treatment of HepG2 cells grown in media either without glucose (NC) or with 0.55 mM fructose(FC1) or with 1 mM fructose (FC2) or with 1 mM fructose + 0.1 µM Insulin (FC3) with AM-HM (75 µg/ml), AM-H (75 µg/ml), AM-C (75 µg/ml), AM-EA (75 µg/ml), AM-B (75 µg/ml) or AM-A (75 µg/ml) for 48 hours. The AM-HM and AM-B reduced the activities of molecular markers of insulin resistance, lipogenesis and inflammation- PI3K (ng/ml), signal transducer and activator of transcription-3 (mAU), mitochondrial target of rapamycin, mTOR (mAU), hypoxia-induced factor, HIF-1α vascular endothelium growth factor(ng/ml), vascular endothelial growth factor, VEGF, (pg/ml) and tumor necrosis factor, TNF-α (pg/ml), a marker of inflammation and insulin resistance in FC1 and FC2. The effect of AM-HM and AM-B was augmented by insulin in FC3. Each value is the mean ± SD of n = 3. *p < 0.05, *vs* control using student-*t*-test.

The level of PI3K was significantly (p < 0.05) decreased in FC1–FC3 groups as compared to NC (Fig. 7a). As compared to FC1, AM-HM1, AM-EA1 and AM-B1 recorded significantly reduced concentrations of PI3K (Fig. 7b). The level of PI3K were significantly elevated in AM-HM2, AM-B2 as compared to FC2 and AM-HM3 as compared to FC3, respectively (Fig. 7c,d).

### Effect of AM-HM on p-try-STAT-3 in HepG2 cell line grown in fructose rich medium

The level of STAT-3 was increased significantly (p < 0.05) in FC1–FC3 groups as compared to NC (Fig. 7e). The level of STAT-3 was significantly (p < 0.05) reduced by the extract and fractions of *A*.*marmelos* in FC2 and FC3 arms (Fig. 7f,h).

### Effect of AM-HM on mTOR in HepG2 cell line grown in fructose rich medium

The level of mTOR was increased significantly (p < 0.05) in FC1–FC3 groups as compared to NC (Fig. 7i). The level of mTOR was significantly (p < 0.05) reduced by the extract and fractions of *A*.*marmelos* in FC2 and FC3 arms (Fig. 7j,l).

### Effect of AM-HM on HIF-1α in HepG2 cell line grown in fructose rich medium

The level of HIF-1α was increased significantly (p < 0.05) in FC1*–*FC3 groups as compared to NC (Fig. 7m). Importantly, the HIF-1α activity was recorded higher in FC2 than either FC1 or FC3. The level of HIF-1α was significantly (p < 0.05) reduced by the extract and fractions of *A*.*marmelos* in FC2 and FC3 arms (Fig. 7n,p).

### Effect of AM-HM on TNF-α in HepG2 cell line grown in fructose rich medium

The concentration of TNF-α was significantly elevated (p < 0.05) in FC1–FC3 groups as compared to NC (Fig. 7q). The level of TNF-α was significantly (p < 0.05) reduced by the extract and fractions of *A.marmelos* in FC2 and FC3 arms (Fig. 7r–t).

### Effect of AM-HM on VEGF in HepG2 cell line grown in fructose rich medium

The VEGF concentration was increased significantly (p < 0.05) in FC1–FC3 groups as compared to NC (Fig. 7u). The level of VEGF was significantly (p < 0.05) reduced by the extract and fractions of *A*.*marmelos* in FC2 and FC3 arms (Fig. 7v–x).

### Effect of AM-HM on fructose uptake and glucose output in isolated murine hepatocytes grown in fructose rich medium

The fructose uptake by isolated murine hepatocytes as indicated by fructose concentration in the media after 6 hours was significantly reduced (p < 0.05) from initial concentration of 181.56 ± 0.12 µg/ml to 129.341 ± 0.172, 135.926 ± 0.172 and 70.926 ± 1.724 µg/ml, in NC, VC and AM-HM, respectively. The glucose concentration was not detected in any of the groups, either at the start or at the end of 6 hours.

## Discussion

The present study was uniquely designed to track the changes in markers of fructose metabolism at different developmental stages (post-weaning, adolescence and adulthood) and compare their presentation pattern. When fructose was provided to developing rats from post-weaning to adolescence stage, firstly, their total caloric intake was higher than age-matched control group, secondly, they ingested fructose to provide for as much as 25% of the total calories consumed. Despite fructose ingestion, the body weight and fasting blood glucose of these animals at adolescence was not starkly different from normal control group. The plasma insulin level was marginally raised, and HOMA-IR values were below that of normal group. However, the results of OGTT evidenced resistance to insulin action as the initial spike in blood glucose levels after glucose ingestion took longer time to revert to baseline than in control group. Similarly, in ITT, after four weeks of fructose intake, the hypoglycemia nadir in 4FDR was at the same time point as 4NDR, albeit significantly steeper, indicating towards the onset of impairment of insulin signaling cascade. The markers of liver health such as weight (55% higher), glycogen content, inflammation, and lipid profile (26% increment in TG level) were severely deranged in the developing rats.

In contrast, at adulthood, there was no difference in the total calories consumed by animals in fructose and normal control arms, but, the animals in former compromised on pellet diet to seek about 17% of the total calories from fructose. The body weight of fructose-fed rats was lower than control group, but the HOMA-IR values were about 7 times higher than control group. There was a concomitant 8-fold leap of in plasma insulin levels and OGTT revealed resistance to insulin action as the blood glucose levels reverted to baseline after 45 min of glucose-feeding, and not after 15 min as seen in control group. In ITT, the simulated hypoglycemia response to insulin, by adult rats was delayed and attenuated, evidencing an insulin resistant state. At adulthood, the weight of the liver was about 20% higher in fructose-fed rats than age-matched control animals, and the level of TG was 4fold higher than control.

The first conclusion that can be drawn from these results is that metabolic alterations in response to fructose ingestion cannot be limited to adulthood, but can be recorded at developing stage, as well. Both the total caloric intake and fraction of calories from fructose are higher in developing rats than adults. It may be hypothesized that central mechanisms that regulate diet preferences and intake, are still in nascent stage in developing animals and not equipped to handle the early onslaught of fructose, or regulate its intake and skewed nutrition choices can derail the metabolic milieu. The hypothesis needs to be supported by clinical data, though preliminary reports have recently surfaced^6^. It may be noted that American Heart Association Nutrition Committee has recommended that women consume no more than 100 kcal/day and men consume no more than 150 kcal/day of added sugar^7^. Further, the Report of the Dietary Guidelines Advisory Committee (DGAC) on the Dietary Guidelines for Americans 2010, has suggested a maximal intake level of 25% or less of total energy from added sugars^8^. Unfortunately, there is severe paucity of such guidelines for developing age-groups. In the condition when such recommendations are formulated, the permissible limit should be much lower than 25% intake of calories from fructose, as the present study indicates that the derangements in metabolic milieu in developing animals were far greater than that recorded in adults consuming lower quantity of fructose.

Secondly, the present results highlight that developing timespan is an important tenure as metabolic derangements due to skewed diet may be more severe and detrimental here. The study II includes animals from 28^th^ day to 84^th^ day of age or from childhood to adulthood stages of lifespan^9^. The results from study I evidence that ingestion of fructose from post-weaning to early adulthood did not translate into raised body weight but positively affected visceral weight (50% higher), at a magnitude that was greater than that recorded in study II at late adulthood (visceral weight gain @ 20%).

There is a strong body of evidence to show that excess visceral adipose tissue is an important determinant of dyslipidemia, insulin resistance and inflammation than body weight^10^. Elsewhere in literature, data from epidemiological study has shown that consumption of sugar-sweetened-beverages by children and adolescents was associated with higher waist circumference, impaired glucose tolerance and visceral adiposity but not subcutaneous abdominal adiposity^11,12^. On the other hand, when men and women were given fructose-sweetened beverages providing 25% of energy requirements for 10 weeks, an increase in intra-abdominal fat by 14.0 ± 5.5% was reported^3^, that appears modest in comparison to effect recorded in children.

It is important to point out that during the early stages of development, the fructose ingestion, lowers the levels of the anorexigenic hormone, leptin, and perhaps initiates re-setting of central mechanisms that can lead to obesity. On the other hand, ghrelin, which is a fast-acting hormone and is reported to be negatively affected by high carbohydrate diet^13^, was also reduced during early stages of development. This may appear contradictory but can be identified as a vital paradigm that can be used to clearly demarcate the condition. As expected, at adulthood, chronic ingestion of fructose had led to leptin resistance and raised levels of orexigenic hormone, ghrelin. Transcription factor hypoxia-inducible factor 1 (HIF-1) represents an acute response to low pO_2_, and activate the transcription of genes involved in an array of signalling events including metabolism^14^.Studies with rat hepatocytes show enhanced DNL supporting a role for low oxygen to regulate hepatic lipid metabolism^15,16^. Thus, targeting HIFs has therapeutic potential for treating liver pathologies^14^. Improved glucose tolerance and insulin signalling in diabetic and non-diabetic mice following treatment with vascular endothelial growth factor (VEGF) inhibitors has also been reported^17^.

The milieu of hepatic enzymes was intriguing as, during developing timespan, the stress of excess fructose availability at the level of hepatocytes, stimulated the glycolytic enzyme (HK), a non-specific, insulin-mediated hepatic enzyme that appears to play a key role in phosphorylating fructose to fructose-1-phosphate in order to maintain energy homeostasis. In the liver, fructose bypasses the two highly regulated steps in glycolysis, glucokinase and phosphofructokinase, both of which are inhibited by increasing concentrations of their byproducts. Interestingly, another enzyme, PFK, that catalyzes unidirectional priming reaction which commits cell to glycolysis and whose rise in activity can be attributed to lowering of ATP/AMP ratio, was raised in adolescent but not adult rats. In contrast, fructose-1, 6-bisphosphatase, which is stimulated by the high energy charge, to bring a net effect of increased gluconeogenesis and glycogen synthesis^18^, was significantly reduced in adolescents but raised in adult rats. The other enzyme, aldehyde dehydrogenase which acts to increase glycerate levels and raise *de novo* lipogenesis and hypertriglyceridemia^19^ was raised in both young developing and adult rats. In concomitance with previous reports in literature for adult animals^20^, it was shown here, that the glucose 6 phosphatase, a gluconeogenic enzyme was also induced by dietary fructose in young developing rats.

In *study I*, it appears that the insulin levels are raised to mediate hepatic metabolism of fructose through the different enzymes, so as to achieve homeostasis, but this inadvertently reflects as raised HOMA-IR. On the other hand, the chronic fructose consumption (8FDR), records significantly high insulin levels which reflect as IR and not homeostatic metabolism of fructose. Thus, it appears, that during developing stages, the fructose consumption stimulates regulatory (PFK) and gluconeogenesis (G6Pase) mechanisms, along with reduction of glycogen synthesis (FBPase, ALDH). The enhancement of both glycolytic and gluconeogenic enzymes appears paradoxical. However, similar metabolic outcomes have been reported elsewhere^20^. It appears that fructose disposal pathways cycle between glycolysis and gluconeogenesis. At the moment this appears futile, but the final verdict on this enigma needs to be reserved till further dissecting studies are undertaken. By adulthood (study II) impairment of energy homeostasis owing to chronic fructose consumption is well-established and evident from disruption of the regulatory mechanisms (PFK) and increment of the gluconeogenic and lipogenic enzymes (FBPase, ALDH), that are antagonistic to the effects of insulin.

The temporal activation of both positive and negative components of the hepatic insulin signaling cascade, i.e., Akt and JNK, respectively, and their associated transcription factors and messengers were also studied. The present study evidences that, fructose ingestion during the post-weaning to adolescent stages of development, promoted compensatory hyperinsulinemia, activated hepatic Akt and inhibited FoxO-1 that together reduced hepatic glucose production, increased hepatic lipid synthesis and initiated hepatostatosis.

Another unique phenomenon associated with hepatic fructose metabolism is that of ATP depletion. Fructose is known to be rapidly phosphorylated to fructose-1-phosphate via fructokinase but cleaves at a slower rate by aldolase B. Thus, fructose behaves as phosphate trap at the expense of depleting ATP stores. Hence, a skewed ratio of AMP/ATP, in favor of former, is indicated by the raised activity of AMP-activated protein kinase. In confirmation, our study shows that AMPK activity was raised during post-weaning to adolescent phase after four weeks of fructose ingestion. Thus, it may be proposed as an important metabolic marker of fructose induced metabolic syndrome in developing animals. The inverse relationship between AMPK activity and hepatic sterol response element binding protein 1 (SREBP-1) is well-established and was also evident here, in both adolescent and adult animals.

To further investigate the molecular and metabolic markers of HepIR, we conducted the *in-vitro* study using HepG2 cells. In the study design, as detailed elsewhere^21^, the cells were grown in fructose enriched media instead of glucose at the concentrations that is reported to be the same as to which hepatocytes are exposed to from portal vein after a fructose-rich meal^22^. Thus, in parallel with the results from *study I*, when HepG2 were grown in fructose enriched media, there was a rise in levels of HK, PI3K-Akt and STAT-3 and reduction in PFK activity and levels of HIF, VEGF and TNF-α in FC1 and FC2. Further, fructose was also rapidly phosphorylated to fructose-1-phosphate and ultimately to glyceraldehyde by the high-activity enzyme- keto-hexokinase (KHK)^23^. This process consumes ATP and rapidly generates the byproduct uric acid that is linked to IR^18^. Since, KHK is neither feedback-inhibited nor allosterically regulated, and bypasses tightly regulated glycolytic checkpoints, the rise in its activity as in FC1 and FC2, creates a sink to facilitate rapid influx of fructose.

For the first time we report here that the early markers for diagnosing fructose induced IR in developing rats are distinct from those at adulthood and show interesting paradigm shift. The liver represents the major sink of fructose, its site for metabolism and generation of multiple effects. Thus, instead of gross markers of metabolic derangements such as HOMA-IR, insulin levels, fasting blood glucose levels or body weight that have been also been demonstrated earlier, to work well in adults^24,25^ we now propose, the estimation of levels of leptin, ghrelin, HK, FBPase and PFK to gain insights in to the true picture of metabolism and diagnose fructose induced HepIR in developing age-groups.

It can now be categorically stated that fructose induced Hep-IR during early developing stages and at adulthood are two different conditions and their diagnosis and management cannot be overlapped. We report here the effects of hydro-alcoholic extract of leaves of *A*.*marmelos* in the two pathological states. The Ru concentration in the extract (AM-HM) was analyzed using in-house developed LC-MS/MS-based method and was estimated to be 1.97 µg/mg. When the extract was orally administered to rats at the dose of 500 mg/kg, the maximum plasma concentration of Ru was 15.3 ± 8.022 ngmL^−1^. In young and adult animals, firstly, the AM-HM reduced the total calories consumed by the rats, and secondly reduced the fraction of calories sourced from fructose to about 20 and 10%, respectively. In both the studies, the weight gain was lowest in AM-HM treated animals. The AM-HM consistently protected against rise in weight of hepatic tissue in both developing and adult rats, clearly indicative of its selective action in mitigating HepIR.

In present study, it appears that AM-HM augments fructose induced gluconeogenesis in 4AMR. In 4AMR, the AM-HM stimulates insulin levels to raise hepatic enzymatic activity (G6Pase, FBPase, HK, ALDH) to tilt the machinery in favour of glucose homeostasis and glycogen formation. The 8AMR recorded marked improvement in insulin signalling as at lower concentration of insulin there was improvement in activities of HK, G6Pase, FBPase, LDH and ALDH, that translated in to lower HOMA-IR.

The AM-HM exerted differential effects on the molecular markers of IR in young and adult fructose-consuming rats. Thus, in fructose-consuming young animals, the AM-HM raised leptin, ghrelin, Akt and STAT-3 levels, to restore homeostatic mechanisms towards glucose sensing, calorie restriction, anorexia and improved insulin downstream signaling. The treatment with AM-HM during this early phase, further, provided impetus to the compensatory cascade by stimulating insulin levels, raising hepatic Akt levels, inhibiting FoxO-1 and increasing total cholesterol levels. In contrast, after chronic fructose intake from post weaning to adulthood, there was no evidence of compensatory cascade attempting to salvage the pathologic state of HepIR in adults. The insulin levels in FDR were about eight times higher than NDR, but did not reflect in either Akt or FoxO-1 levels, suggesting a severe state of resistance at the level of post-receptor downstream signalling. The treatment with AM-HM, reduced insulin demand, restored downstream signaling via Akt and FoxO-1 to reduce glycogen levels. Further, mitigation of AMPK activity and elevation of SREBP-1 by AM-HM in fructose drinking animals belonging to developing age-group but not in adults, is indicative of its ability to favorably restore AMP/ATP ratio in former but not in latter. As SREBP1c is an important transcription factor that regulates the expression of hepatic gluconeogenesis and *de novo* lipogenesis, the evidence from the present study gives credence to the protective action of AM-HM at developing stages.

The availability of hepatic GLUT2 protein was also reduced by AM-HM to restrict fructose influx into hepatocytes. In adult animals who were chronic consumers of fructose, AM-HM acted by reducing insulin requirement and improving downstream signaling. It also reduced leptin resistance, pro-inflammatory markers, and hepatic glucose transporters. In corroboration, with the reported literature, present study shows that 4AMR and 8 AMR recorded reduction in HIF and VEGF that was raised in 4FDR and 8 FDR, respectively.

Thus, till date it was widely accepted that the high-fat-diet induced insulin resistance and metabolic disorders but present experimental data supports previous reports that high-fructose diet is equally detrimental in causing metabolic disorders^26^. Further, the extract (AM-HM) and its butanol fraction (AM-B) performed consistently to revert the skewed paradigms. Based on the data the butanol fraction of AM-HM can be further developed for its preventive and therapeutic action in mitigating fructose induced HepIR.

## Methods

### Authentication of leaves

The leaves of A.marmelos were locally collected, identified and authenticated by Principal Scientist, National Bureau of Plant Generic Resources (Indian Council of Agricultural Research), New Delhi, India, and a specimen has been saved as voucher (NHCP/NBPGR/2014–6).

### Preparation of extract

The leaves were washed, shade dried, powdered and exhaustively cold macerated with ethanol: water (1:1 v/v), filtered and concentrated (AM-HM).

### Preparation of fractions

The hydroalcoholic extract (AM-HM) was fractionated using n-hexane (AM-H), chloroform (AM-C), ethyl acetate (AM-EA), n-butanol (AM-B). The aqueous phase from all steps of partitioning was pooled as an aqueous fraction (AM-A).

### LC-MS/MS of extract and fractions

The 4000Q-TRAP triple quadrupole, tandem mass spectrometer (AB Sciex, Foster City, CA, USA) coupled with ultra-high-performance liquid chromatography (UHPLC, Accela Thermo Fisher Scientic, Waltham, MA, USA) with auto-sampler and online vacuum degasser was used. The tandem mass spectrometer and UHPLC were controlled by Analyst software, version 1.4.2(AB Sciex, Foster City, CA, USA) and ChromQuest software, version 4.5 (Thermo Fisher Scientic, Waltham, MA, USA), respectively.

### Optimization of MS detection and chromatographic conditions

The analytical separation of Rutin (Ru) was optimized using Purospher star C18 Column (50 × 4.6 mm, 3.5 µm, Merck, Germany). The mobile phase consisted of (A) water with 0.1% formic acid and (B) methanol with 0.1% formic acid. The gradient elution for run time of 5 min was, 20% B (0–0.5 min), increased to 80% B (0.5–1 min), maintained till 3 min, decreased to 20% B at 3.5 min and maintained till 5 min at a flow rate 1 ml/min.

Electrospray ionization in a negative mode was applied using Turbo Ionspray source (AB Sciex, Foster City, CA, USA). The full scan mass spectra and fragment ion spectra of Ru were obtained by flow infusion analysis (FIA). For optimization, Ru and Probenecid were pumped (Harvard Company, Reno, NV, USA) at a flow rate of 5 µl/min. Quantification was performed using multiple reaction monitoring (MRM) mode based on the molecular/fragment ion transitions for Ru (609.1/300.1) and IS (283.910/240). Source dependent parameters were optimized by flow infusion analysis (FIA): gas 1(40 psi), gas 2(60 psi); curtain gas (30 psi); collection gas (6 psi); ion spray voltage (4500 V) and temperature (450 °C). Compound dependent parameters like Declustering Potential (DP), Entrance Potential (EP), Collision Energy (CE) and Cell Exit Potential (CXP) were manually optimized for Ru as 133, 10, 53 and 14 V, respectively. For IS, the compound dependent parameters: DP, EP, CE and CXP were manually optimized as 100, 70, 10, 22 and 3 V, respectively.

### Calibration standard

The blank plasma (Blood Bank, All India Institute of Medical Sciences, New Delhi) was spiked with standard Ru (3.9–500 ngmL^−1^) to achieve LOD as 0.7911 and LOQ as 2.397 ngmL^−1^.

### Sample preparation for standardization of extract and fractions

For quantification of Ru, in AM-HM and its fractions, standard Ru was mixed with IS, and analysed.

### Pharmacokinetic study of AM-HM

Human plasma was mixed with standard Ru (500, 250, 125, 62.5, 31.25, 15.6, 7.5, 3.91 µgmL^−1^), and IS and injected.

### Method validation for pharmacokinetic study of AM-HM

The LC-MS/MS method was validated according to the currently accepted US Food and Drug Administration (FDA) Bioanalytical Method Validation Guidelines^27^.

### Calibration curve, linearity and sensitivity of pharmacokinetic study of AM-HM

Five concentrations of Ru between 3.9–500 ngmL^−1^ were plotted and a correlation coefficient (R^2^) of 0.99 or better was selected. The lower limit of quantification (LLOQ) was defined as the concentration with accuracy of 80–110% and precision (% CV) < 20%.

### Accuracy and precision of pharmacokinetic study of AM-HM

Intra and inter-day assay precisions were determined as %CV (Coefficient of Variance).

### Absolute recovery and matrix effect of pharmacokinetic study of AM-HM

The recovery of Ru was determined by standard addition method. Three pre-quantified (7.8, 15.6 and 500 ngmL^−1^) concentrations were selected.

### Animals

Male Albino Wistar rats (250 ± 10 g) were used in accordance with the guidelines of CPCSEA and the protocol was approved by Institutional Animal Ethics Committee (DIPSAR/IAEC/2015-II/07).

### Drug administration and blood sample collection

The food deprived (12 h) animals were administered AM-HM (500 mgkg^−1^, po) and under Ketamine (80 mgkg^−1^, ip) anaesthesia, blood samples (0.8 mL) were collected from the tail vein at 0.08 min, 1, 2, 4 and 24 h and stored at −80 °C until analysis.

### Sample preparation for plasma quantification

The rat plasma was spiked with IS, vortexed, sonicated and analyzed using LC-MS/MS.

### Experimental design to study onset of fructose induced insulin resistance during developing and adulthood stages in rats

As per approved protocol (DIPSAR/2015-II/6-7), Wistar albino rats (male, 4 weeks old, 50–55 g) were housed under standard laboratory conditions with pellet diet (M/S Pranav Agro Industries Ltd., India) and drinking water or fructose, *ad libitum*.

#### Study I

Weaned animals (4 weeks old) were randomly divided into three groups (n = 6 each)- normal control (4NDR, chow + drinking water), fructose control (4FDR, chow + fructose,15%), and treatment (4AMR, chow + fructose,15% + AM-HM 500 mg/kg/d, po) for 4 week study duration.

#### Study II

Weaned animals (4 weeks old) were randomly divided into three groups (n = 6 each)- normal control (8NDR, chow + drinking water), fructose control (8FDR, chow + fructose,15%), and treatment (8AMR, chow + fructose,15% + AM-HM500 mg/kg/d, po) for 8 week study duration.

### Measurement of feed and fructose/water intake, body weight and caloric intake

Intake of pre-measured feed and fructose/water by the animals over 24 hr duration was recorded.

The daily body weight chart of each animal was maintained. The total caloric intake (=kcal of metabolizable energy/g diet intake + kcal of energy/gm fructose intake) of each group was calculated.

### Measurement of FBG, OGTT, HOMA IR and ITT

The FBG of fasted animals was measured by glucometer (ACCU-CHECK^®^ GO, Hoffmann-La Roche Ltd). Whole body insulin sensitivity was assessed by Oral Glucose Tolerance Test (OGTT)^28^ at different time points (0, 15, 30, 60, 90, 120 min). Homeostasis model assessment- index of insulin resistance was calculated as:

$${\rm{HOMA}}\,{\rm{IR}}={\rm{glucose}}\,{\rm{level}}\,(\mathrm{mg}/\mathrm{dl})\times \mathrm{insulin}/\mathrm{405}.$$

Insulin tolerance test (ITT) was performed according to Lehnen *et al*., 2010. Briefly regular insulin (Huminsulin Eli Lilly & company India Pvt Ltd, India) (0.75 U/kg) was injected subcutaneously to animals and blood samples were collected from tail vein at 0 (just before insulin injection), 30, 60, 120 min (after insulin the injection) for glucose estimation using glucometer (Hoffmann-La Roche Ltd, India).

### Blood and viscera collection

Upon study completion, blood was collected by cardiac puncture, and plasma separated. The viscera (heart, kidney, liver) from sacrificed animals, were weighed digitally. The hepatic tissue was sectioned and appropriately stored.

### Biochemical estimations

Fasting serum insulin and plasma leptin and ghrelin concentrations, was assessed (SPI bio, Germany). Lipid profile including total cholesterol, triglyceride, high density lipid (HDL), low density lipid (LDL) and very low-density lipid (VLDL) were measured in serum (Grenier Diagnostic, Germany). Phosphatidylinositol-4,5-bisphosphate 3-kinase (PI3K, Bioassay Laboratory Technology, China), insulin (Ray Biotech, USA), phospho-Akt (Ray Biotech, USA), Resistin-Like Molecule (RELM, Ray Biotech, USA), phosphorylated-signal transducer and activator of transcription-3 (p-tyr-STAT-3, Ray Biotech, USA), uric acid (Abcam Lab, USA), serum glutamic-oxaloacetic transaminase (SGOT, Erba Lab, India), serum glutamic-pyruvic transaminase (SGPT, Erba Lab, India), hypoxia-inducible factor 1-alpha (HIF-1α, Ray Biotech, USA), vascular endothelial growth factor (VEGF, Krishgen Biosystems, India) and tumor necrosis factor (TNF-α, Krishgen Biosystems, India) were measured in liver sample.

### Glycogen and hepatic enzyme estimation

Glycogen content in liver was estimated according to standard protocol^29^. The liver homogenate was assessed for glucose-6-phosphatase (G6Pase), fructose-1,6-bisphosphatase (FBPase)^30^, hexokinase (HK)^31^, lactate dehydrogenase (LDH)^32^, aldehyde dehydrogenase (ALDH)^33^, Alkaline Phosphatase (ALK, Accurex Biomedical, India) and Phosphofructokinase (PFK, KinesisDx, USA).

### Western blot analysis

The liver homogenate was prepared as per standard protocol and Glut2 protein was immunodetected after overnight incubation with specific primary Rabbit polyclonal antibodies (ab95256, 1: 10,000) and corresponding secondary antibody-HRP-conjugated Goat Anti-rabbit IgG (ab6721,1:20, 000) and assessed by densitometry (G: Box, Syngene, Frederick, USA).

### Hepatic histology

For histopathological assessment by trained pathologist who was blinded for the study, fixed liver samples (10% formalin) were sectioned (4 µm) and stained with haematoxylin and eosin (H&E).

### Hepatic immunohistochemistry for GLUT2 protein

Paraffin embedded liver sections were subjected to heat-induced antigen retrieval and incubated in rabbit anti-rat GLUT2 (1:100 dilution) as per standard protocol.

### Transmission electron microscopy of hepatic tissue

In accordance with standard procedure, fixed hepatic tissue was cut using an ultra-microtome contrasted with uranyl acetate and lead citrate and viewed (TECNAI 2000 KV TEM, Fei, Electro Optics, Netherlands).

### *In vitro* study design: IR induction by fructose in HepG2 cells

The human hepatocellular carcinoma cell line (HepG2) was acquired from National Centre for Cell Sciences (NCCS), Pune, India, and grown under standard culture conditions. HepG2 cells were seeded (1 × 10^5^ cells/well/2 ml) and grown for 48 h either in – DMEM-glucose (NC), DMEM-glucose + 0.55 mM fructose(FC1), DMEM-glucose + 1 mM fructose (FC2) or DMEM-glucose + 1 mM fructose + 0.1 µM Insulin (FC3).

### Cell treatment and sample collection

The cells in the different groups (FC1–FC3) were treated with either DMSO (0.1% v/v, VC1–VC3), AM-HM (75 µg/ml, AM-HM1–AM-HM3), AM-H (75 µg/ml, AM-H1–AM-H3), AM-C (75 µg/ml, AM-C1–AM-C3), AM-EA (75 µg/ml, AM-EA1–AM-EA3), AM-B (75 µg/ml, AM-B1–AM-B3) or AM-A (75 µg/ml, AM-A1–AM-A3) and the supernatant and cell lysates were collected and preserved at −80 °C for analysis.

### Biochemical estimation

The cell lysate was used for estimating glycogen concentration, enzyme activities (Hexokinase, HK; Aldehyde dehydrogenase, ALDH; Ketohexokinase, KHK; Phosphofructokinase, PFK) using respective ELISA kits (Sincere, China). The phosphatidylinositol-4,5-bisphosphate 3-kinase (P13K), signal transducer and activator of transcription 3 (STAT-3), mammalian target of rapamycin (mTOR) and hypoxia-inducible factor 1-alpha (HIF-1α, Ray Biotech, USA), were estimated in cell lysate. In supernatant tumor necrosis factor (TNF-α, Krishgen Biosystems, India) and vascular endothelial growth factor (VEGF, Krishgen Biosystems, India), were estimated.

### Statistical analysis

Results are expressed as the mean ± standard deviation of the mean and statistically analyzed by one-way analysis of variance (ANOVA) followed by Tukey’s multiple comparison test. Statistical analysis was performed using, Graph Pad prism 5.0 ver for Windows (Graph Pad Software, San Diego, CA, USA). A value of p < 0.05 was considered statistically significant. The data of plasma concentration versus time was subjected to a known compartmental pharmacokinetic using WinNonlin software (Pharsight) to obtain an estimate of various pharmacokinetic parameters.

## Electronic supplementary material

Supplementary Information

Supplementary Information
