# Supplementary figures and images for: Molecular and Metabolic Markers of Fructose Induced Hepatic Insulin Resistance in Developing and Adult Rats are Distinct and Aegle marmelos is an Effective Modulator
Source: Sci Rep. 2018 Oct 29;8:15950. doi: 10.1038/s41598-018-33503-x (PMC6206063; doi:10.1038/s41598-018-33503-x)

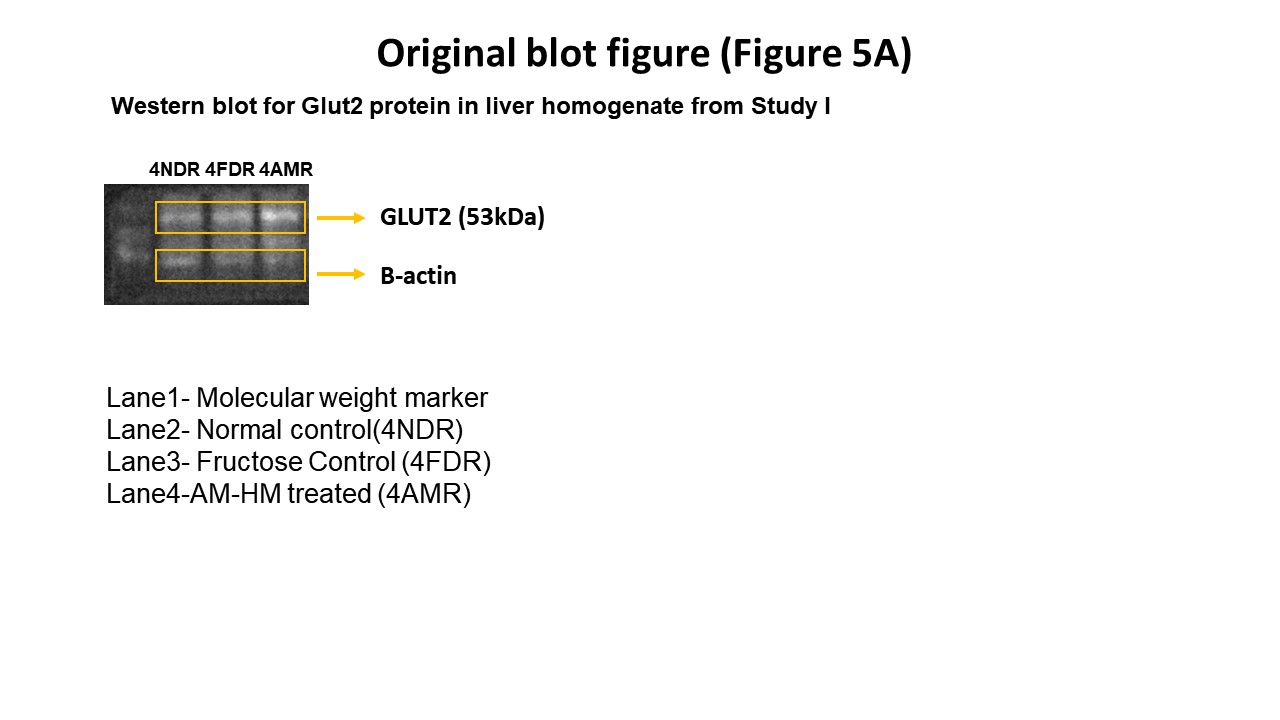

Supplement: Supplementary file 1 — Supplementary Information [file 41598_2018_33503_MOESM1_ESM.jpg]

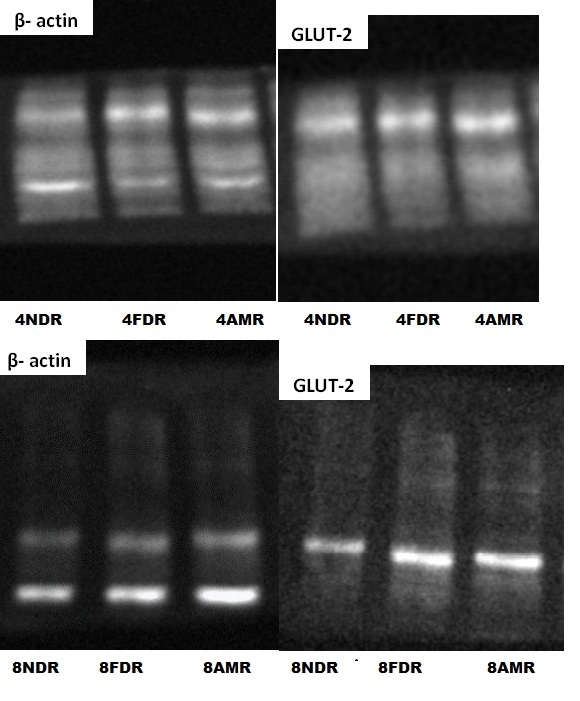

Supplement: Supplementary file 2 — Supplementary Information [file 41598_2018_33503_MOESM2_ESM.jpg]
